# Editorial: Unraveling the role of receptor signaling in tumorigenesis and cancer therapy

**DOI:** 10.3389/fcell.2026.1770251

**Published:** 2026-02-26

**Authors:** Srijan Acharya, Girijesh Kumar Patel, Mohammad Aslam Khan, Amanda M. Clark, Sirin Saranyutanon, Shashi Anand

**Affiliations:** 1 Department of Pharmacy and Pharmaceutical Sciences, St. Jude Children’s Research Hospital, Memphis, TN, United States; 2 Department of Biotechnology, Motilal Nehru National Institute of Technology, Allahabad, India; 3 Department of Pathology, University of Pittsburgh, Pittsburgh, PA, United States; 4 Department of Anatomical Pathology, Faculty of Allied Health Sciences, Burapha University, Chonburi, Thailand; 5 Cancer Center and Research Institute, University of Mississippi Medical Center, Jackson, MS, United States; 6 Department of Cell and Molecular Biology, University of Mississippi Medical Center, Jackson, MS, United States

**Keywords:** CD44, Ect2 RhoGEF, frizzled (Fzd), integrin β4 high expression cancer, lysine acetylation (LysAc), pancreatic malignancies, Rab5B, receptor signaling in cancer

Receptor-mediated signaling serves as a cornerstone of tumor initiation, progression, and therapeutic response, making it one of the most transformative areas of modern cancer research. This is exemplified by the pathogenic role of aberrantly activated receptors, including receptor tyrosine kinases, G-protein–coupled receptors (GPCRs), and immune checkpoint receptors. Cancer cells manipulate receptor-mediated signaling to continuously reinterpret their habitats and selectively thrive under stress. Shaped by mechanical cues, developmental history, epigenetic landscapes, cellular architecture, and immune pressures, this dynamic signaling sustains survival, promotes invasion, and enables resistance to treatment.

Multiomics approaches and integrative analyses have enabled the identification of molecular signatures that drive oncogenic transcriptional programs, reshape the tumor microenvironment, and promote metastatic dissemination. These tools are revealing an unprecedented level of complexity in receptor-ligand interactions and signaling plasticity across diverse cancer types. Consequently, these insights are not only redefining the molecular taxonomy of tumors but also accelerating the development of next-generation therapeutics, ranging from allosteric inhibitors and antibody-drug conjugates to chimeric antigen receptor (CAR) T-cells and bispecific antibodies to improve clinical management. As we continue to dissect how receptor signaling networks are rewired during tumor evolution and therapy resistance, a systems-level understanding will be essential for designing durable, patient-tailored interventions.

The articles in this Research Topic showcase how diverse scientific approaches are converging on a unified insight: cancer signaling is best understood as a dynamic, multilayered network rather than an isolated pathway. A recurrent theme in this Research Topic is the ability of tumors to convert physical cues into biochemical programs. Mechanosensing receptors such as integrin β4 translate extracellular matrix interactions into oncogenic cascades that promote epithelial–mesenchymal transitions (EMT), migration, and therapeutic resistance (Huang et al.). Upon detachment from their structural anchors, these receptors function as signaling scaffolds that cooperate with binding partners to amplify AKT, mitogen-activated protein kinase (MAPK), and Src - focal adhesion kinase (Src-FAK) pathways, creating a signaling architecture optimized for metastatic dissemination. This work underscores a growing realization that mechanical cues are central to how malignant cells acquire invasive behavior.

During oncogenesis, key signaling networks acquire another dimension from developmental biology. Pathways that guide embryonic morphogenesis, including Hedgehog, transforming growth factor-β (TGFβ), Notch, and KRAS-driven circuits, are frequently redeployed in pancreatic malignancies. As described by Swain et al., these developmental programs create lineage-specific vulnerabilities, drive cellular plasticity, and foster therapeutic-resistant niches. Tumors, in essence, borrow the logic of early organ formation to construct microenvironments favorable to growth, thereby evading constraints normally imposed on adult tissues.

Moreover, even the most sophisticated signaling pathways depend on the broader regulatory environment. For example, lysine acetylation of histones and non-histone proteins can powerfully shape how cells perceive and respond to receptor-derived stimuli. Li and Xue highlight how altered acetylation states in digestive tract cancers restructure chromatin accessibility, alter protein stability, and recalibrate downstream pathway output. This regulatory layer shows that similar receptor signals can drive distinct transcriptional and phenotypic consequences across tissues and disease contexts.

Adding further nuance, alternative splicing generates receptor isoforms with distinct signaling capabilities. CD44, a multifunctional adhesion and signaling receptor long recognized for its complexity, exemplifies this characteristic. Variant isoforms, such as those containing v3, v6, or v9, can alter ligand affinity, change receptor-partner interactions, and reshape downstream stress responses. Yanova et al. demonstrate how these isoforms regulate apoptosis, autophagy, and ferroptosis, thereby shaping chemoresistance across multiple cancer types. Here, RNA processing becomes a driver of signaling plasticity, enabling tumors to adapt rapidly under therapeutic pressure.

Among key GPCRs, the Frizzled (FZD) family of Wnt receptors remains mechanistically challenging, with unresolved questions about ligand specificity, receptor conformation dynamics, and intracellular coupling. Martinez-Marin et al. review structural and functional advances that clarify how FZD receptors shape canonical and non-canonical Wnt signaling outputs, and highlight receptor complexity as a longstanding barrier to drug development. As structural resolution improves, opportunities are emerging to rationally target Wnt signaling, one of cancer’s most notoriously difficult pathways, with improved precision.

Within the cell, cytoskeletal regulators act as crucial integrators, linking receptor activation to changes in cell shape, polarity, and motility. The epithelial cell transformation factor 2 (ECT2)–RhoA–Rho-associated protein kinase (ROCK) axis, as described by Sheng et al., illustrates how alterations in polarity and contractility amplify oncogenic signaling and promote invasion in gynecologic malignancies. The cytoskeleton, in this sense, is not merely an architectural scaffold but a dynamic signaling hub that modulates how tumors respond to extracellular cues.

Importantly, signaling adaptation is also shaped by receptor routing and trafficking. Endosomal signaling and receptor internalization determine the strength, duration, and spatial organization of pathway activation, and thus can contribute to therapeutic resistance and metastatic propensity. Consistent with this, Zhu et al. show that altered RAB5B (an early endosomal guanosine triphosphatase (GTPase)) expression correlates with key malignant features, including immune infiltration, genomic instability, metastatic potential, and responsiveness to immunotherapy. In parallel, immune checkpoint receptor rewiring under therapeutic pressure highlights signaling plasticity, enabling tumors to remodel immune pathways and evade immune control. Together, these observations highlight that receptor signaling plasticity is not just confined to membrane activation events but also extends across intracellular trafficking compartments and tumor–immune interfaces.

Advances in cryo-electron microscopy, single-cell multiomics, and live-cell imaging continue to deepen our understanding of complex signaling events and enabling the development of more effective therapies. There is growing interest in targeting post-translational regulators, splicing programs, mechanotransduction elements that convert mechanical force into biochemical signals, and trafficking nodes, either alone or in combination with canonical receptor inhibitors. These strategies recognize that durable therapeutic responses require not only shutting off a receptor but also anticipating adaptive rewiring within signaling networks.

The work presented here reflects a rapidly evolving field that continues pushing the boundaries of our understanding about intercellular communication within the tumor microenvironment. By revealing the interconnected and context-dependent nature of receptor signaling networks, these studies can provide a foundation for developing more effective, adaptable, and personalized cancer therapies. As our knowledge deepens, so does the potential to reshape the therapeutic landscape—not only by targeting a single pathway, but by also engaging multiple dynamic signaling networks that sustain malignancy. Collectively, the contributions in this Research Topic present a cohesive story of receptor signaling and highlight its multifaceted, context-dependent role in human malignancies.

